# Non-operative management of blunt splenic trauma: evolution, results and controversies

**DOI:** 10.1590/0100-6991e-20202777

**Published:** 2021-04-24

**Authors:** JOSÉ DONIZETI MEIRA, CARLOS AUGUSTO METIDIERI MENEGOZZO, MARCELO CRISTIANO ROCHA, EDIVALDO MASSAZO UTIYAMA

**Affiliations:** 1 - Hospital das Clínicas da Universidade de São Paulo, Departamento de Cirurgia - São Paulo - SP - Brasil

**Keywords:** Angiography, Splenectomy, Embolization, Therapeutic, Abdominal Injuries, Conservative Treatment, Angiografia, Esplenectomia, Embolização Terapêutica, Traumatismos Abdominais, Tratamento Conservador

## Abstract

The spleen is one of the most frequently affected organs in blunt abdominal trauma. Since Upadhyaya, the treatment of splenic trauma has undergone important changes. Currently, the consensus is that every splenic trauma presenting with hemodynamic stability should be initially treated nonoperatively, provided that the hospital has adequate structure and the patient does not present other conditions that indicate abdominal exploration. However, several topics regarding the nonoperative management (NOM) of splenic trauma are still controversial. Splenic angioembolization is a very useful tool for NOM, but there is no consensus on its precise indications. There is no definition in the literature as to how NOM should be conducted, neither about the periodicity of hematimetric control, the transfusion threshold that defines NOM failure, when to start venous thromboembolism prophylaxis, the need for control imaging, the duration of bed rest, and when it is safe to discharge the patient. The aim of this review is to make a critical analysis of the most recent literature on this topic, exposing the state of the art in the NOM of splenic trauma.

## RESUMO

O baço é um dos órgãos mais frequentemente afetados no trauma abdominal contuso. Desde os trabalhos de Upadhyaya, o tratamento do trauma esplênico vem sofrendo importantes modificações. Atualmente, é consenso que todo trauma esplênico que se apresenta com estabilidade hemodinâmica pode ser tratado inicialmente de forma não operatória, desde que o serviço possua estrutura adequada e o paciente não apresente outras condições que indiquem exploração da cavidade abdominal. Entretanto, vários tópicos permanecem controversos no que diz respeito ao tratamento não operatório (TNO) do trauma esplênico. A angioembolização esplênica é uma ferramenta de grande auxílio no TNO, porém não há consenso sobre suas indicações precisas. Não há uma definição na literatura a respeito da forma como o TNO deve ser conduzido, tampouco a respeito da periodicidade do controle hematimétrico, do limiar de transfusão que define falha do TNO, de quando iniciar a profilaxia contra tromboembolismo venoso, da necessidade de exames de imagem de controle, do período de repouso no leito, e de quando é seguro indicar alta hospitalar. O objetivo desta revisão é analisar de forma crítica a literatura a respeito desse tema, expondo o estado da arte no TNO do trauma esplênico.

## INTRODUCTION

The spleen is one of the most frequently affected organs in abdominal trauma^1^ ^,^ ^2^, presenting injuries in up to 16% to 23.8% of polytraumatized patients, with a mortality rate of 9.3%, mainly due to associated injuries and delayed treatment^3^ ^,^ ^4^. Currently, most cases are treated nonoperatively^5^. The advantages of this modality include reduction of costs, of non-therapeutic laparotomy rates, of intra-abdominal complications, of blood component transfusion, of morbidity and mortality^5^ ^-^ ^8^. Furthermore, splenic preservation avoids exposure of patients to overwhelming post-splenectomy infection, a potentially fatal condition caused by encapsulated organisms in splenectomized patients^9^.

Splenic lesions are most often classified according to the American Association for the Surgery of Trauma (AAST) Organ Injury Scale^12^. Grade I and II lesions have a risk of rebleeding below 20%. Grades III, IV and V lesions present a risk greater than 20%, reaching 50% when associated with contrast extravasation, and reaching up to 70% when there is extensive hemoperitoneum (blood in the periesplenic recess, parietocolic gutters and pelvis)^10^. In two recent retrospective studies, the frequency of grade I to V injuries were, respectively, 8-13%, 22-37%, 25-39%, 16-25%, and 6-9%^10^ ^,^ ^11^.

Although it is the modality of choice for most cases, nonoperative management is not without flaws. The rate of failure of nonoperative management (NOM) is currently lower than 10%^6^ ^,^ ^13^ ^,^ ^14^, but widely variable according to the trauma classification, reaching 75% of failure of NOM for patients with grade V splenic trauma^15^. Failure of NOM is associated with higher mortality^16^ ^,^ ^17^, highlighting the importance of early identification of the cases in higher risk for this condition. At-risk patients may undergo closer monitoring or even early splenic angioembolization^5^. 

Although indications for NOM of splenic trauma have been extensively studied in the literature, there are few studies defining the evolution of the selected patients, and there are few widely used protocols for the conduction of NOM^15^. Several topics related to the follow-up of these patients are still controversial. These include the frequency of clinical reevaluations and laboratory tests, the duration of patient monitoring, transfusion thresholds that would indicate intervention (surgery or angiography), time to iniciate venous thromboembolism (VTE) prophylaxis, the time of bed rest and hospital stay, the need for immunization after spleen embolization and after extensive splenic injury submitted to NOM, the indication of control imaging and outpatient follow-up after hospital discharge^5^ ^,^ ^7^ ^,^ ^18^.

The aim of this review is to critically analyze the evolution of NOM of splenic trauma, it’s predictors of failure, and the main controversies in the literature so far.

Until the 1980s, splenic lesions were routinely treated with splenectomy^19^. According to Upadhyaya, it was mistakenly believed that the spleen had no function, that nonoperative treatment was lethal, that there was an imminent risk of rupture if the organ was preserved, and that the spleen could not be sutured^9^. Morris and Bullock were the first to show the protective function of the spleen against infections in the early twentieth century^20^, demonstrating that splenectomized rats had higher postoperative mortality than those undergoing simulated operations, attributing the difference in mortality to sepsis induced by bacillus that caused caused plague in rats. Several years later, King and Schumacker published a series of cases of post-splenectomy fulminant sepsis caused by encapsulated bacteria in children undergoing splenectomy^21^, leading to a discussion about the harm of traumatic asplenia also in adults^22^ and the potential benefit of preserving this organ.

The first reports of NOM for splenic injuries came from studies with children^19^ ^,^ ^23^, and since then the incidence of splenectomy in the context of splenic trauma has dropped significantly^24^. Given the good results with this new modality, adult patients began to be contemplated with NOM from the 1980s onwards^25^ ^,^ ^26^.

It is currently agreed that every hemodynamically stable patient may initially undergo NOM if some resources are available in the hospital^5^. NOM is feasible in hospitals where full-time surgeons, blood banks, easy access to imaging methods (computed tomography is mandatory), and intensive care unit are available^27^. However, even these patients may have associated conditions that require surgical exploration, such as hollow viscera lesions. According to the literature, associated injuries occur in 36% of patients with blunt splenic trauma, 9.6% of wich are from hollow viscera^28^ ^-^ ^30^. The operative care of splenic injury associated with hollow viscus injury affords contemporaneous operative care of the splenic injury, whether by splenectomy, splenorraphy, or simply by packing the spleen with hemostatic gauze. However, lesions that result in perforation and leakage of these viscera contents are rare in blunt abdominal trauma, with incidence around 0.3%^29^, which guarantees certain safety in the adoption of NOM. With regard to penetrating mechanisms, few studies address the NOM of splenic lesions, and currently there is insufficient evidence to suggest a broad incorporation of this practice safely into victims of penetrating spleen injury.

As NOM was increasingly instituted, cases of failure of NOM began to become more evident. In order to identify which patients were most at risk, several studies sought to determine the predictors of failure of NOM.

### Predictors of failure of nonoperative management

Most patients with low-grade splenic injuries (I to III according to AAST) are successfully submitted to NOM. However, even patients with high-grade lesions (IV-V AAST) may initially undergo NOM, provided they are hemodynamically stable^12^ ^,^ ^31^. It is noteworthy that even patients who meet the criteria for NOM (hemodynamic stability, absence of lesions requiring surgical exploration, and available resources) may evolve with failure of NOM. It is evident that one of the main current questions regarding the approach to NOM in splenic trauma is: what is the profile of the patient with high risk for NOM failure? 

Several factors have been studied in the literature, including age, degree of splenic injury^15^, Injury Severity Score (ISS) values, hemoperitoneum volume, vascular abnormalities, need for transfusions and hematimetric levels^32^.

In a study conducted by Olthof et al.^32^, the Delphi method was used to reach consensus among surgeons and interventional radiologists regarding NOM and its prognostic factors. The results of this study indicated that it is necessary to consider a higher probability of failure of NOM for patients aged 40 years or older, with Injury Severity Score (ISS) equal to or greater than 25, and for those with grade III-V splenic lesion. The risk of failure of NOM is also higher when there are associated liver injuries^33^. World Society of Emergency Surgery Guidelines state that there is strong evidence that age greater than 55 years, elevated ISS, and moderate to severe splenic injuries are independent predictors of failure of NOM^12^.

In a more recent review, Olthof et al.^13^ states that the degree of splenic injury, the presence of a large hemoperitoneum, contrast extravasation at admission, a high ISS value (≥25), systolic hypotension on admission, transfusion of more than 1 packed red blood cells, and the presence of traumatic brain injury increases the likelihood of failure of NOM. However, there is no specific definition regarding hemoperitoneum volume that would increase the rate of failure of NOM. Although there are no data regarding adult population, the absence of detectable hemoperitoneum by Point-Of-Care Ultrasonography has been evaluated in a recent study with 292 patients, which demonstrated that the presence of negative FAST predicts NOM success in the pediatric population^34^. In a study evaluating NOM for blunt abdominal trauma victims with multiple intra-abdominal solid organ injuries, patients that failed NOM had significantly higher serum lactate levels, hematocrit drop greater than 20% within the first hour, and higher degree of solid viscera injury^35^.

Just as important as knowing the population at risk for failure of NOM is to know when the failure usually occurs. A 2005 study^36^ showed that 40% of failure of NOM cases occur within 4 to 8 hours of patient admission, and stated that 88% of failure of NOM occurred within the first 5 days of observation, and 93% in the first week. Two recent studies^15,37^ that involved over 10,000 patients showed that 85% to 95% of failure of NOM cases occurred within the first 3 days of observation. The most recent study from 2008 also showed that monitoring for an additional 2 days made it possible to diagnose 1.5% more failure of NOM. Therefore, it is recommended that patient observation should be performed for 3 to 5 days, allowing identification of up to 97% of failure of NOM.

### Angiography

Splenic embolization has been considered a tool to decrease failure of NOM cases. In a 1995 study^38^, the authors performed systematic angiography in every patient undergoing NOM and showed success with proximal splenic artery embolization in patients with contrast extravasation on angiography. Since then, several studies have analyzed the role of angiography in reducing failure of NOM rates and, consequently, in increasing cases of splenic preservation, especially when early performed^39^. Embolization has been applied in several trauma centers due to high risk conditions for failure of NOM, such as high-grade splenic injuries (grades IV and V), vascular abnormalities (arteriovenous fistulae, contrast leakage and pseudoaneurysms) and large hemoperitonium^5^ ^,^ ^32^. It is important to outline that, as an invasive procedure, it may present complications. Thus, it is necessary to identify the cases that really benefit from embolization angiography in order not to expose patients to unnecessary risks.

There is controversy when analyzing the meaning of tomographic blush in the main references on the subject. The presence of blush is of great importance in the Western Trauma Association guidelines, as it indicates angioembolization in grade III^40^ lesions, while it is much less important according to the EAST guidelines, which advocate the presence of blush should not only be considered iteself as an indication of angioembolization, but the patient’s entire clinical condition should be considered^5^. In the guidelines of the World Society of Emergency Surgery^12^, blush is only used to indicate arteriography in grade I to III trauma. Some authors point out that the absence of blush does not exclude the presence of active bleeding in patients with high-grade trauma (IV or V)^41^. In a recent study, the presence of blush increased the need for arteriography by 6 times in patients with grade I-III trauma^42^. In 2017, on the other hand, a new review on the subject by Olthof^13^ suggests that contrast extravasations smaller than 1.0 to 1.5 cm do not require angioembolic intervention^43^ ^,^ ^44^.

Once decided by splenic embolization, the results regarding proximal (splenic artery trunk) or distal (also called selective) embolization must be analyzed. Proximal embolization is usually used when bleeding is diffuse, when the patient’s hemodynamic condition is borderline, or when vascular anatomy is unfavorable. In cases where bleeding is focal, distal embolization is used. It is noteworthy that, although there are no prospective studies, proximal embolization is faster, and has lower failure and complication rates compared to distal^45^. However, Olthof et al. suggests that the distal should be the preference, because if it fails there would still be the possibility of a new attempt of embolization, this time proximal^13^.

The main complications of splenic embolization are splenic infarction, abscess, hyperthermia and hyperalgesia without associated splenic infarction. Such complications may occur in up to 47% of cases^14^. 

A large national retrospective study^46^, enrolling over 37,000 patients, performed in the USA revealed that splenic artery embolization had the higher rates of infection at 1 year when compared with NOM and operative management. The etiology of this increased risk is unclear, but it may be related to some ischemic areas in the spleen after embolization. This study didn’t evaluate the difference between proximal and distal splenic artery embolization in the infection rates.

### Controversies

Although the spleen is one of the organs most commonly affected in blunt abdominal trauma, NOM is the method of choice for most patients, and angiography has a clear role in reducing the failure of NOM rates, there are still several controversies in the literature. 

While the indication of NOM is well established, and its main predictors of failure have been extensively studied, there are not many published norms related to the evolution of these patients. In other words, although there is consensus on which patients should undergo NOM, little is known about how to follow them once the nonoperative strategy has been established.

How should hematimetric control be performed? How long should patients be kept at rest and when should they return to activities? When to start venous thromboembolism (VTE) prophylaxis? Is there a need for control imaging? What is the impact of splenic embolization on its immune function? These are some topics on which there is no consensus yet. Although extremely important for the proper follow-up of patients undergoing NOM, there is no strong evidence to suggest any specific recomendation. Thus, it is necessary to perform a critical analysis of the works already published.

Most studies referring to hematimetric control discuss the frequency with which hemoglobin and hematocrit levels should be obtained. In fact, there is currently not enough evidence to guide a specific regimen^5^. In a consensus of experts^32^, most agreed that it was necessary to collect hemoglobin or hematocrit every 4 or 6 hours within the first 24 hours of onset of NOM or until level stability. After this period, it was recommended that the measurements should be performed once or twice a day. Despite being a controversial concept, studies seem to agree that hematimetric stability is defined by a fall of less than 0.5mg/dL in two consecutive measurements, and that the measurements of hematimetric levels should be frequent in the first day of NOM (at least every 6 hours), and further apart in the following days (once or twice every day).

The relationship between early patient mobilization and failure of NOM is discussed as well. In a recent study, Teichman et al.^47^ compared a three-day absolute rest regimen with an early deambulation based on hematimetric stability for patients with splenic or hepatic trauma. The authors concluded that there was a decrease in the hospitalization time of the group with early deambulation without increasing the failure of NOM rate. The study by London et al.^48^ presented a similar conclusion. Thus, it is currently recomended that these patients should not be kept in bed rest.

Regarding the beginning of VTE prophylaxis, there is no consensus either. The incidence of thromboembolic complications in patients with solid organ injuries can reach 4.5%, resulting in morbidity^49^ ^,^ ^50^. As with non-operative treatment of other abdominal organs, there is some concern in initiating prophylaxis due to the risk of rebleeding and failure of NOM^51^. However, studies have shown that it is safe to introduce VTE chemoprophylaxis within the first 48h-72h of hospital admission^18^ ^,^ ^49^ ^,^ ^51^ ^,^ ^52^ without increasing the incidence of failure of NOM^52^. Joseph et al.^49^ observed a tendency towards a higher incidence of thromboembolic complications in patients receiving late prophylaxis (after 72h), although this difference was not statistically significant. The relationship between VTE prophylaxis and higher failure of NOM or rebleeding rate was also not observed in other studies^50^ ^,^ ^53^. A recent study^54^ involving more than 36,000 blunt trauma patients undergoing NOM showed that early introduction (within 48h of the injury) of VTE chemoprophylaxis was associated with lower rates of deep vein thrombosis and pulmonary thromboembolism, without causing significant difference in the need for blood transfusion, the incidence of failure of NOM or mortality. Thus, despite being a controversial topic, the literature recommends early initiation of chemoprophylaxis in the first 48-72h, considering the magnitude of the splenic lesion and the risk of patient bleeding individually to decide the ideal moment to initiate VTE prophylaxis.

Another aspect discussed in the NOM of splenic lesions concerns the threshold of transfused blood units that would define failure of NOM and, therefore, the need for surgical intervention or embolization. There is no consensus in the literature about this value. Fodor et al. indicates that transfusion of 2 or more red blood cell concentrates should already be indicative of failure of NOM^18^. In this case, NOM should only be continued if the cause of the need for transfusion is related to other lesions; however, this decision is based on clinical judgment, as in practice it is difficult to establish the cause of the need for transfusion when there is more than one possible focus of bleeding. Few studies show results related to this topic, and the decision to change conduct considering a possible failure should not be based on an arbitrary blood transfusion value. We consider that this decision should be based on other clinical and laboratory aspects, and often supported by imaging results.

There is also discussion about the need for control imaging after the onset of NOM of splenic injury. It is currently accepted that vascular abnormalities may appear later than the first CT scan. One study showed that pseudoaneurysms can appear even in grade II and III^55^ injuries on a control tomography between 1 and 8 days of trauma in 15% of the cases, half of which evolved with spontaneous pseudoaneurysm occlusion, without the need of any intervention. As such, repeat imaging appears to be an unnecessary practice because it did not influence the treatment^18^. However, it is noteworthy that data regarding the long term evolution of these patients are lacking, especially regarding the need for angioembolization of the lesions identified in the control CT^56^ ^,^ ^57^. In a recent review^57^ ^,^ ^58^, patients submitted to NOM were followed with routine imaging examination (either ultrasound or CT scan), between zero (within 24h) and 11 days from the initial CT scan. Fifty-five exams (96,4%) had no new significant findings, and the other two had more abdominal fluid compared with the initial CT, but both had an uneventful further course. None of the CTs revealed delayed pseudoaneurisms or arterio-venous fistula. Therefore, routine follow-up imaging appears to have limited therapeutic advantages. Indication for follow-up imaging should be based on clinical deterioration, and CT scan should be used as the preferred imaging modality.

Asplenia is a condition associated with immune deficiency and can result in fulminant infections with encapsulated germs. Despite being a rare condition - occurs in only about 2% -, patients submitted to splenectomy are at risk of developing these infections^57^. They usually occur within the first 2 years and are associated with a 70% mortality^57^ ^,^ ^59^. To prevent this complication, vaccines should be administered 2 weeks before or 2 weeks after splenectomy for a better immune response. In the context of trauma, vaccination is usually performed 2 weeks after the surgical procedure. It is currently recommended that these patients should receive pneumococcal, Haemophilus influenzae type B and meningococcal vaccine, and annual influenza vaccination. One of the current controversies is whether splenic embolization is associated with decreased immune function and thus the need for immunization as in splenectomized patients. A recent meta-analysis specifically studied this issue^60^. Of the 12 included studies, 11 demonstrated preserved splenic function after angioembolization in both adults and children. Thus, there is currently no evidence to recommend routine vaccination of these patients, and each case should be analyzed individually.

## CONCLUSION

NOM of blunt splenic injuries can be indicated in every hemodynamically stable patient, provided there are adequate resources in the hospital and there are no associated lesions that require surgical exploration. Once decided for NOM, it is imperative to identify the main predictors of failure. Although not contraindicating NOM, the presence of these factors should alert the physician, motivating him to closely monitor the patient. Unfortunately, there is no consensus on various aspects of follow-up of these patients, and protocols are not uniform in most services. Therefore, the treatment should be individualized according to the best available evidence.
